# Nitrous Oxide the Safest Volatile Anesthetic

**Published:** 1916-08

**Authors:** 


### NITROUS OXIDE THE SAFEST VOLATILE ANESTHETIC.

---

There is no longer any question, if Dr. Charles S. Skaggs is right—as set forth in his paper in *The Lancet-Clinic* for September 18, 1915, page 247—that nitrous oxide, when administered in association with oxygen, is the safest of our volatile anesthetics, provided it is given by an experienced anesthetist. Dr. Skaggs does not mean to imply that nitrous oxide is the anesthetic of choice for all operations, but he does believe that with this agent the patient can thus, for a short period of time, be anesthetized with less danger.

Ether, declares Dr. Skaggs, is contra-indicated as an anesthetic for tuberculosis patients; indeed, patients suffering from organic diseases of the lungs and kidneys, as well as from severe suppurative conditions, asthma, empyema or diabetes, frequently do not respond well to ether or chloroform. Nitrous oxide and oxygen, on the contrary, can be used in conditions like those named with comparative safety.—*Clinical Medicine.*

